# Evaluation of Immediate Implantation and Provisionalization Combined with Guided Bone Regeneration by a Flap Approach in the Maxillary Esthetic Zone: A Retrospective Controlled Study

**DOI:** 10.3390/ma14143874

**Published:** 2021-07-12

**Authors:** Zhenya Su, Yuan Chen, Maoxia Wang, Anchun Mo

**Affiliations:** State Key Laboratory of Oral Diseases, Department of Implant Dentistry, West China School/Hospital of Stomatology, Sichuan University, Chengdu, China; suzhenya@stu.scu.edu.cn (Z.S.); chenyuanwestchina@stu.scu.edu.cn (Y.C.); 2018324035051@stu.scu.edu.cn (M.W.)

**Keywords:** immediate provisionalization, GBR, anterior maxilla, dental implants, flap operation

## Abstract

The aim of this research was too compare the thickness change of labial contour and bone tissues, as well as some biological complications of immediate implantation with and without immediate provisionalization for a single anterior maxilla presenting a vertical defect on labial bone with the need of guided bone regeneration (GBR) by a flap approach. A total of 40 single implants were placed in 40 patients into fresh extraction sockets of the anterior maxilla with a vertical defect on the labial bone (<4 mm). Simultaneously, GBR was conducted at the sites by a flap approach, and the implants were given immediate or delayed provisionalization. The thickness change of bone tissues during six-month evaluation and labial contour during three and six-month follow-up were measured. Complications such as implant and restoration survival rates, infection as well as wound exposure were also evaluated at six months postoperatively. After six months, the mean thickness losses in labial bone were 0.9040, 0.8050, 0.7165, 0.5285 and 0.5335 mm at five different sites in immediate provisionalization group, and 0.8780, 0.8605, 0.7560, 0.5900 and 0.6300 mm, respectively, in delayed provisionalization group, showing no significant difference between the groups at all measurement sites. Although the labial contour changes of the two groups were similar at most sites, the values at 1 and 2 mm above the implant neck remained significantly lower in the immediate provisionalization group at three and six months postoperatively. No complications occurred during the follow-up time. Based on the limitation of this study, the immediate implantation combined with GBR, flap operation and immediate provisionalization obtained acceptable outcomes for a single anterior maxilla with vertical defect on the labial bone, but more long-term research with a larger sample are urgently needed in the future.

## 1. Introduction

With the increasing expectations for simplified treatment and improved aesthetics, immediate implantation and provisionalization has become a well-accepted strategy, especially for the anterior maxilla with a thick labial plate (>1 mm) [1,2]. Since the treatment concept of immediate implantation and provisionalization has been established, it aimed at a better preservation of the peri-implant bone and mucosa to achieve long-term success and esthetic results [3]. Compared with conventional protocol with the need of 2–3 consecutive surgeries, it had less tissue damage, scarring and loss at the implantation site [4]. Clinical research has demonstrated that immediate implantation shows similar success rates as delayed placement in healed sockets, and the immediate provisionalization of a single maxillary anterior offered high implant survival rates of 96% to 100% [5,6,7,8,9]. In addition, the instant placement of a provisional crown provided a mechanical support to the mid-facial gingiva and papilla, thus extra soft tissue surgery may be eliminated [10,11]. Until now, scholars have recommended this treatment in patients with an intact labial bone, because immediate implantation by a flap was proven to induce a significant bone resorption as well as a high risk of mid-facial recessions [12]. Clinically, however, approximately 87% of human labial bone walls were thin (<1 mm) or even lacking, which required additional guided bone regeneration (GBR), and usually in this situation, early implantation with submerged healing by a flap operation may be a more conventional selection [13,14]. To date, clinical research on immediate implantation and provisionalization in such challenging cases has never been reported. Thus, this prospective study was the first to evaluate whether immediate implantation and provisionalization exhibited different effects from immediate implantation and delayed provisionalization in the maxillary anterior zone when simultaneous GBR procedure was conducted utilizing a flap approach, to provide a full protocol in clinics.

## 2. Material and Methods

### 2.1. Patient Selection

A total of 40 patients who received single tooth extraction, immediate implantation, flap operation, simultaneous GBR, immediate or delayed provisionalization in the maxillary anterior region at West China Hospital of stomatology, Sichuan University, China from 2020 to 2021 were included in this study. Patient selection was conducted on the basis of the following criteria.

Inclusion criteria:

1. Males and females aged 18 years at least
2. Single tooth with indications for extraction in the maxillary anterior zone (incisor and canine) with both neighboring teeth present
3. Presence of a vertical defect less than 4 mm on the labial bone around the neck of the inserted implant
4. At least 4 mm apical bone allowing implantation with the minimum primary stability of 35 Ncm

Exclusion criteria:

1. Acute infection around the implant sites or uncontrolled periodontal diseases
2. Any systemic contraindication to the implantation
3. Psychiatric problems, alcohol, tobacco (>20 cigarettes per day) or drug abuse
4. Pregnancy or lactation
5. Insufficient oral hygiene, occlusal instability or severe bruxism
6. Unwillingness for follow-up examination

This study was conducted according to the Helsinki declaration of 1975, as revised in 2013. All included patients have signed an informed consent form prior the study. Ethics approval was obtained from the local ethical committee (No: WCHSIRB-OT-2020-146).

### 2.2. Surgical Procedures

**Figure 1:**
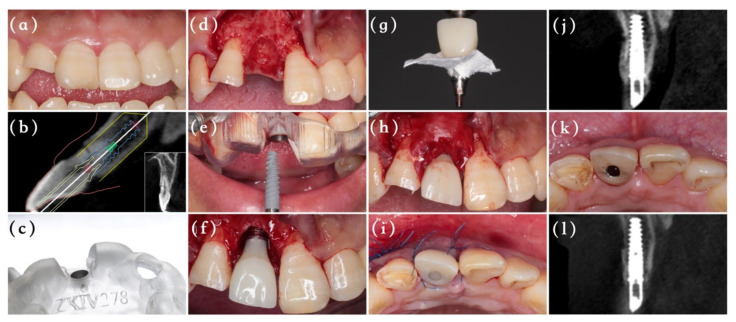
The surgical flow and follow-up of Group A. (**a**) Baseline clinical situation of the hopeless tooth #8. (**b**) Digital implant planning. (**c**) The fully surgical guide (**d**) Tooth extraction of #8. (**e**) Immediate implant placement guided by template. (**f**) The labial boney defect was observed following immediate provisionalization. (**g**) Collagen membrane was immobilized by temporary restoration. (**h**) The bone graft substitute was covered with a absorbable membrane. (**i**) Flap closure and sutured. (**j**) CBCT taken immediately after surgery. (**k**) Occlusal view at 6 months. (**l**) CBCT image 6 months after surgery.

**Figure 2:**
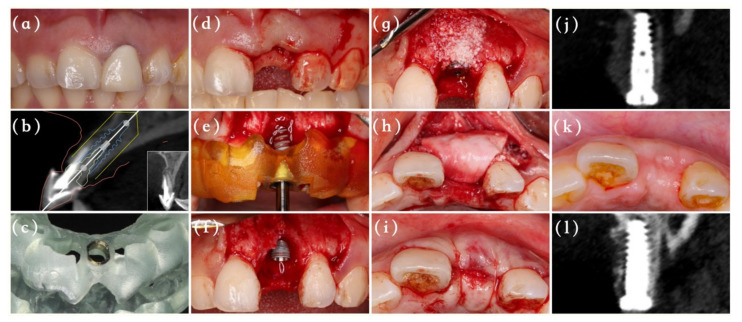
The surgical flow and follow-up of Group B. (**a**) Baseline clinical situation of the hopeless tooth #9. (**b**) Digital implant planning. (**c**) The fully surgical guide (**d**) Tooth extraction of #9. (**e**) Immediate implant placement guided by surgical template. (**f**) The labial boney defect was observed following immediate implantation. (**g**) Bone graft material was placed into the defect area and the healing abutment was screwed onto the implant. (**h**) The bone graft substitute was covered with an absorbable membrane. (**i**) Flap closure and sutured. (**j**) CBCT taken immediately after surgery. (**k**) Occlusal view at 6 months. (**l**) CBCT image 6 months after surgery.

The included patients received periodontal scaling preoperatively, and 2 g of amoxicillin was administrated one hour before the surgery. All surgical and restoration procedures were undertaken by the same experienced surgeon (MA), as depicted in Figure 1 and Figure 2. After administration of a local anesthesia with 4% solution of articaine, the surgeon extracted the tooth as atraumatically as possible to preserve the labial bone plate, and then a full-thickness triangular flap was elevated directly. The whole surgery procedure from the initial drilling to the implant insertion was conducted with the aid of a digital template using NobelActive Guide Drilling Kit (Nobel Biocare AB, Gothenburg, Sweden). All implants used here were NobelActive (Nobel Biocare AB, Gothenburg, Sweden) with an insertion torque of at least 35 Ncm. After implant placement, a periodontal probe was used to measure the vertical defect of labial bone around the implant neck.

**Figure 3:**
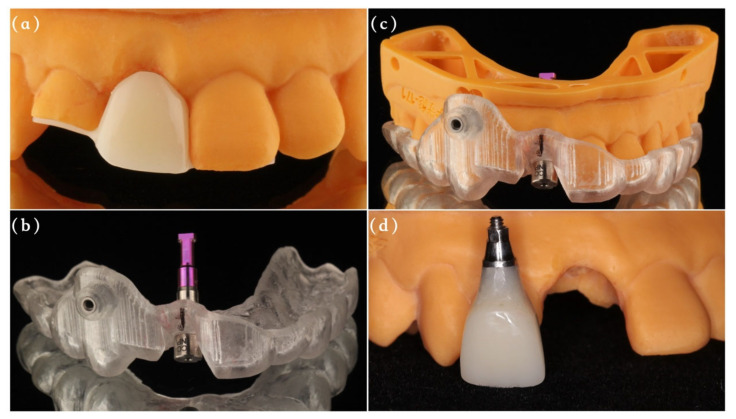
The fabrication process for provisional restoration. (**a**) The temporary crown with resin wings was made before the surgery. (**b**) Analogs was fixed on the surgical template by the guided cylinder with pin. (**c**) Implant analogs were inserted to the predetermined position into resin models with the help of full-guided templates. (**d**) The temporary abutment was matched and a screw-retained temporary restoration with an appropriate emergence profile was prefabricated.

Group A (immediate provisionalization): Before the surgery, an implant analog was inserted to the predetermined position into patients’ models with the help of full-guided template and guided cylinder pin, and then the temporary prosthesis was made with a resin crown and Nobel temporary abutment (Nobel Biocare AB, Gothenburg, Sweden) (Figure 3). Following implantation, the jumping gap and vertical bone defect surrounding the implant were carefully filled with deproteinized bovine bone particles (Bio-Oss, Geistlich Pharma AG, Wolhusen, Swizerland), and numerous cortical perforations were made properly. Afterwards, an absorbable collagen membrane (Bio-Gide, Geistlich Pharma AG, Wolhusen, Swizerland) was fixed by 6/0 unabsorbable sutures and the prefabricated screw-retained temporary crown to cover the bone grafts with an overlap of 2 mm. Any centric or eccentric contact of the provisional crown was avoided. A releasing incision was made in the periosteum to make the flap elastic, and the mucosa was sutured around the temporary crown by 6/0 unabsorbable sutures to achieve tension-free primary repair. After 6 months, the patients received final restorations.

Group B (delayed provisionalization): The GBR and suture procedures were as the same as group A, with the only difference being that the absorbable collagen membrane was only fixed by 6/0 unabsorbable sutures. A standard healing abutment (Nobel Biocare AB, Gothenburg, Sweden) rather than a temporary crown was screwed into the implant to obtain the submerged healing. With the bone and soft tissue maturation 6 months after implant placements, the surgeon conducted secondary-stage surgeries for each patient. After a temporary restoration phase of 3 months, the final restoration was finished.

Postoperatively, the patients were instructed to take the antibiotics (1 g amoxicillin) three times a day for 3 days and rinsed with 0.2% chlorhexidine mouthwash twice daily for 2 weeks. The sutures were removed 2 weeks following the surgery. Patients needed to come back for recall as required, and the time for the first visit, the time immediately after surgery and the recall at 3 and 6 months were set as T0, T1, T2 and T3 respectively.

### 2.3. Labial Bone Measurements

**Figure 4:**
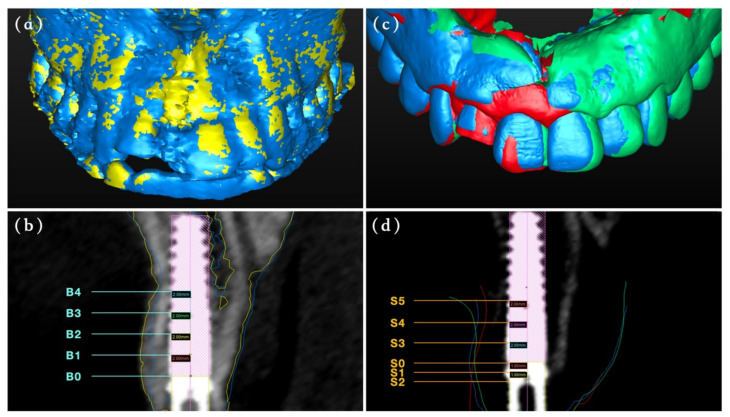
Contour change analysis. (**a**) Showing the STL (converted from Dicom in SimPlant^®^ software) superimposition results for different morphology on two time points (T1: yellow, T3: blue). (**b**) The thickness of labial bone was measured from the labial outlines of the bone (T1: yellow, T3: blue) to the implant (B0-B4). (**c**) Showing the STL superimposition results for different morphology on three time points (T0: red, T2: green, T3: blue). (**d**) The thickness of labial contour was measured from the labial outlines (T0: red, T2: green, T3: blue) to the implant (S0–S5).

The cone beam computed tomography (CBCT) scans at T1 and T3 were obtained to evaluate the labial bone thickness change surrounding the implant using CBCT machine (3D Accuitomos, Morita, Kyoto, Japan) with the same parameters (acceleration voltage, 90 kVp; beam currency, 5 mA; acquisition 17.5 s; voxel size 0.25 mm; slice thickness 0.25 mm and field of view 140 mm × 100 mm). Firstly, the 6-month CBCT in Digital Imaging and Communication in Medicine (DICOM) format was converted to surface tessellation language (STL) file in SimPlant^®^ software (Materialise, Leuven, Belgium), and then it was superimposed with DICOM files at T1 to measure the bone thickness changes during the healing stage. The horizontal distance between the radius of the interior contour of the implant and the labial bone outline (including bone grafts) in the bucco-oral cross-sectional plane perpendicular to the implant was measured along the implant neck (B0) towards the apical at 2, 4, 6 and 8 mm (B1–B4) (Figure 4a,b). The measurements were taken 3 times by one independent calibrated examiner (CY) who did not participate in any treatment process, and the average value was recorded accordingly.

### 2.4. Labial Contour Measurements

An experienced surgeon used an intraoral scanner (3Shape, Copenhagen K, Denmark) to evaluate the labial contour alterations at T0, T2 and T3. The STL files of all data were introduced into SimPlant^®^ to measure the contour changes, with the T0 model as baseline. To achieve the best superimposition, the data were matched by automated tools at first and then aligned manually using anatomic landmarks. The horizontal distance between the radius of the interior contour of the implant and the labial outer contour in the bucco-oral cross-sectional plane perpendicular to the implant was measured at the implant neck (S0), 1 and 2 mm below (S1–S2), as well as 2, 4, 6 mm above (S3–S5) (Figure 4c,d). The measurements were repeated 3 times to eliminate potential inaccuracy.

### 2.5. Biological Complications

Surgical complications such as implant and restoration survival rates, infection as well as wound exposure were recorded with a follow-up half a year after implantation. The success criteria for implantation suggested by Buser et al., was that the implant should be in its original position without peri-implant inflammation and radiolucency [15].

### 2.6. Statistical Analysis

Statistical analysis was conducted using SPSS 21.0. software (SPSS Inc., Chicago, IL, USA), with the patient as the unit. Continuous variables were presented as the mean and standard deviation (SD), while categorical variables were described by frequency and percentage. The Shapiro–Wilk test was used to verify the normal distribution. Accordingly, values were compared with independent t-test for continuous variables and Chi-squared test for categorical variables. The level of scientific significance was set at *p* < 0.05.

## 3. Results

### 3.1. Details of the Included Patients

The background information of the included patients was described in Table 1. A total of 40 patients with a mean age of 39.8 (range from 21 to 62) received 40 single-tooth replacements in the maxillary anterior zone by means of immediate implantation in combination with simultaneous GBR using a flap approach. Afterwards, 20 patients accepted immediate provisionalization, while the other 20 received delayed provisionalization. As shown, the most common implant site was the central incisor and no significant difference was found in patient age and gender (*p* > 0.05).

### 3.2. Thickness Change of Labial Bone Tissues Based on CBCT Scan

Table 2 depicted the thickness change of labial bone plate around the implant. As shown, the values at T1 were similar between the two groups, ensuring the reference level was comparable. With T1 model as the baseline for analysis, the mean thickness reduction of labial bone plate from the implant neck to 2,4,6,8 mm above were 0.9040, 0.8050, 0.7165, 0.5285 and 0.5335 mm, respectively, in the immediate provisionalization group, and 0.8780, 0.8605, 0.7560, 0.5900 and 0.6300 mm, respectively, in the delayed provisionalization group during the six-month observation, with no significant difference between the groups. Over time, the bone thickness reduced in both two groups, and the largest resorption was observed at the implant neck with a mean loss of 0.9040 mm in the immediate provisionalization group and 0.8780 mm in the delayed provisionalization group.

### 3.3. Thickness Change of Labial Contour Based on Intraoral Scanner

More details about the labial contour dimension at different measurement sites and time intervals were presented in Table 3. Taking the T0 functioning as the baseline, the labial contour changes at all measurement sites were negative, indicating the resorption at three and six-month timepoints. At three-month evaluation, the changes at implant neck, 1 and 2 mm below, as well as 2, 4, 6 mm above were −0.1155, −0.1175, −0.1280, −0.0995, −0.0445 and −0.0140 mm, respectively, in immediate provisionalization group, and −0.1935, −0.5620, −0.5980, −0.1450, −0.0925 and −0.0825, respectively, in delayed provisionalization group. Six months later, the corresponding values were −0.1085, −0.1190, −0.1310, −0.1420, −0.0680 and −0.0350 mm, respectively, in immediate provisionalization group, and −0.3335, −0.5060, −0.6565, −0.1505, −0.0990 and −0.0885 mm in delayed provisionalization group. At three and six-month follow-up, the immediate provisionalization group suffered less labial contour collapse, with statistical significance at 1 and 2 mm below the implant neck, while at other measurement sites, the difference was insignificant. Besides, the dimension of labial contour only decreased gradually within the first three months, and then remained virtually stable from T2 to T3.

### 3.4. Biological Complications

Based on the criteria for successful osseointegration, the implant survival rates in both two groups were 100% during the follow-up time. Besides, all 20 temporary crowns remained stable, resulting in 100% survival rate at a half year post-operatively. No complication including infection and wound exposure occurred in the two groups during the six-month evaluation.

## 4. Discussion

Over the decades, implant dentistry has tended to reduce the treatment time, optimize the aesthetics, and improve patient satisfaction. As a result, immediate implantation and provisionalization has become an alluring choice, which can not only simplify surgical procedures and provide instant esthetics, but also maximize the preservation of bone and gingival architecture in the anterior esthetic region [16]. Some studies have reported that immediate implantation and provisionalization had high implant survival rate [17]; however, as suggested by ITI consensus in 2018, it was not a scientifically validated protocol and cases should be selected carefully to reduce the potential risks [18]. In the event of a vertical defect presenting on the labial bone, the early implantation combined with simultaneous GBR, flap operation and delayed provisionalization after soft tissue healing was usually regarded as a safer strategy by scholars, but this still lacked sufficient clinical evidence [19]. This study proposed a new method that the tooth extraction, flap surgery, implantation, GBR and immediate provisionalization were completed in one surgical session for a single maxillary anterior with a vertical defect on the labial bone, aiming to simplify the treatment protocol under the premise of guaranteeing the therapeutic effect. The thickness change of labial contour and bone tissues, as well as biologic complications after immediate implantation, were compared between immediate and delayed provisionalization groups.

With regard to the bone tissue alterations, both immediate and delayed provisionalization groups exhibited horizontal resorption on the labial bone plate after six months of healing when compared to the T1 model. Although it failed to show significant difference between the groups at all measurement sites, more bone resorption was noted in delayed provisionalization group, probably related to its extra surgical procedures. Similar to our results, the study by Slagter et al. also reported that the immediate provisionalization group obtained a slightly thicker layer of labial bone, but the different point was that they did not conduct GBR by a flap way at the implant sites [20]. In fact, simultaneous GBR has been regarded as a routine therapy in cases with local bone defect showing a favorable morphology around implants, and in this situation, submerged healing was a more common choice to reduce the exposure of bone graft materials and improve the bone formation [21]. However, in the present study, immediate provisionalization group exhibited less bone resorption at all measurement sites than delayed provisionalization group, although the difference was insignificant. Previous studies have proven that several factors including the use of immediate provisionalization, flap or flapless technique, and the presurgical thickness of buccal bone plate had great effects on the labial bone resorption following immediate implantation [22]. Compared with a clinical study by Yang et al. that showed that the mean bone loss at the implant neck was 1.1000 mm at six months postop in cases with 0–0.5 mm thick labial walls, a slightly lower bone resorption of 0.9040 mm was obtained in our study, which may be facilitated by the overbuilding GBR using a flap operation [13]. Although many studies reported that less bone resorption occurred by flapless implantation, this conclusion was still contradictory [23,24]. Conventionally, a flapless operation is recommended in sites with thick bone wall due to the difficulty in bone augmentation and increased risk for vestibular perforation [25]. It seemed that the temporary crown improved the bone formation, and a possible explanation may be the fact that the temporary crown contributed to stabilize the collagen membrane for GBR, and its support for soft tissues may also reduce the compression to the bone grafts inside. During a six-month follow-up, the most obvious bone resorption was observed at the implant neck, whether in immediate or delayed provisionalization group, indicating more attention should be paid to this site when immediate implantation and simultaneous GBR were conducted in the maxillary anterior zone.

As introduced in previous studies, the stability of facial contour following implantation was one of the most critical parameters for esthetic evaluation in the anterior region [26,27]. Compared with the preoperative data at T0, the changes of labial contour at T2 and T3 were presented as negative values in both two groups, indicating a thickness reduction at all measurement sites during the healing period. Results from this study show that immediately restored implants were accompanied by a better preservation of labial contour at 1 and 2 mm above the implant neck, which was in agreement with previous research [28]. Compared with a stock healing abutment, the proper emergence profile of provisional restorations may contribute to support the peri-implant tissues [29,30,31,32]. It has been reported by Chu et al. that the anatomic temporary prothesis can increase the soft tissue thickness by 0.5–1 mm and reduce the horizontal collapse to less than 0.2 mm [33,34]. A similar conclusion was also obtained by Crespi et al., showing a better volume maintenance in immediate provisionalization group [35]. As a consequence, there have been many investigators advocating the application of immediate provisionalization in esthetic zones for not only patient pleasure but also its advantage in terms of the soft tissue contour [9]. In the present study, we used a new method introduced by Zhang et al. for immediate provisionalization [36]. Different from the traditional provisional crown fabrication procedure with the need of impression or intraoral fabrication, the pre-made temporary crown can be fixed on the implants immediately after the surgery, thus reducing the chair side time and simultaneously providing more time to optimize the profile design. A concave profile design for provisional crowns was adopted here to avoid any pressure on bone and soft tissues as depicted by Gomez-Meda et al., and any centric or eccentric contact was avoided during the whole implant healing period [37].

Although some researchers proposed that the wound healing may be poor when immediate implantation was combined with simultaneous GBR [38], all patients in our study obtained a good primary wound closure after implantation without any infection or wound exposure. In fact, the key points such as incision design, tension-reducing operation and elaborate suture were important to avoid these postoperative complications. In this study, a periosteal splitting was conducted towards the marginal bone crest to release the muscle tension, and in this way, the wound can be closed passively. Too date, no biologic complicates such as implant and restoration failures have occurred in both two groups, but the follow-up time was too short and a long-term observation is urgently needed.

The combination of immediate implantation, simultaneous GBR, flap operation and immediate provisionalization is recommended in our study; however, some limitations have to be addressed. Firstly, the number of included cases was very limited with too short a follow-up time. Furthermore, we lacked full-scall measurements for each patient, such as the mid-facial mucosa level, aesthetics score and patient-reported satisfaction. In the future, we will keep following the labial bone and contour changes of the included patients in this study for five years after the implantation. Secondly, some measurement errors for bone tissues caused by CBCT artifacts were unavoidable; thus, it was hard to evaluate the thickness when the bone was thin. Finally, some potential factors may also influence the labial contour and bone tissue following implantation and GBR procedure such as the gingival biotype and osseous structures; thus, more research is needed to explore it.

## 5. Conclusions

In this retrospective study, it could be concluded that the thickness change of labial bone tissues was similar between the immediate and delayed provisionalization groups, while the immediate provisionalization group obtained more stable labial contour at 1 and 2 mm below the implant neck at three and six-month evaluations. Based on this short-term follow-up, the immediate implantation and provisionalization obtained acceptable therapeutic outcomes in the maxillary anterior region even though GBR was conducted by a flap approach, but vital fundamentals like case selection, treatment planning and postoperative manipulation should be considered thoughtfully to reduce the high risks potentially. In the future, more studies are urgently needed to demonstrate the treatment efficacy of this procedure.

## Figures and Tables

**Table 1:** Details of patients and treatments characteristics.

Group	No. Patients (Ratio)	Sex		Age		Implant Sites			
		Male (Ratio)	Female (Ratio)	Mean ± SD	Range	11/21	12/22	13/23	Total
Total Patients	40 (-)	17 (43%)	23 (57%)	39.80 ± 10.72	21–62	21	10	9	40
Group A (immediate provisionalization)	20 (50%)	6 (30%)	14 (70%)	39.35 ± 13.11	21–62	12	5	3	20
Group B (delayed provisionalization)	20 (50%)	11 (55%)	9 (45%)	40.20 ± 8.33	27–53	9	5	6	20

**Table 2:** Thickness changes in labial bone tissues at different levels for each group (Group A = immediate provisionalization; Group B = delayed provisionalization).

Items	Groups	T1 Mean ± SD	*p* Value	T3-T1 Mean ± SD	*p* Value
B0	Group A	3.93 ± 0.713	0.16	0.90 ± 0.68	0.909
	Group B	3.56 ± 0.90		−0.87 ± 0.73	
B1	Group A	4.00 ± 0.86	0.212	−0.80 ± 0.64	0.799
	Group B	3.76 ± 0.86		−0.86 ± 0.72	
B2	Group A	4.00 ± 0.86	0.479	−0.71 ± 0.84	0.875
	Group B	3.79 ± 0.97		−0.75 ± 0.72	
B3	Group A	3.62 ± 0.97	0.529	−0.52 ± 0.84	0.817
	Group B	3.44 ± 0.72		−0.59 ± 0.82	
B4	Group A	2.89 ± 1.14	0.599	−0.53 ± 0.57	0.640
	Group B	2.73 ± 0.67		−0.63 ± 0.71	

**Table 3:** Thickness changes in labial contour compared with the pre-surgical status at different levels for each group (Group A = immediate provisionalization; Group B = delayed provisionalization).

Items	Groups	T0 Mean ± SD	*p* Value	T2-T0 Mean ± SD	*p* Value	T3-T0 Mean ± SD	*p* Value
S2	Group A	2.67 ± 0.78	0.699	−0.12 ± 0.71	0.035 *	−0.13 ± 0.98	0.037 *
	Group B	2.76 ± 0.68		−0.59 ± 0.64		−0.65 ± 0.40	
S1	Group A	2.88 ± 0.79	0.835	−0.11 ± 0.77	0.047 *	−0.11 ± 0.47	0.011 *
	Group B	2.93 ± 0.81		−0.56 ± 0.58		−0.50 ± 0.43	
S0	Group A	2.61 ± 0.56	0.982	−0.11 ± 0.61	0.665	−0.10 ± 0.48	0.121
	Group B	2.61 ± 0.53		−0.19 ± 0.51		−0.33 ± 0.41	
S3	Group A	2.14 ± 0.11	0.508	−0.09 ± 0.55	0.803	−0.14 ± 0.52	0.957
	Group B	2.11 ± 0.08		−0.14 ± 0.59		−0.15 ± 0.46	
S4	Group A	2.69 ± 1.52	0.975	−0.04 ± 0.81	0.826	−0.06 ± 0.78	0.881
	Group B	2.68 ± 0.87		−0.09 ± 0.53		−0.09 ± 0.47	
S5	Group A	2.93 ± 1.21	0.642	−0.01 ± 0.89	0.761	−0.03 ± 0.89	0.814
	Group B	2.76 ± 1.06		−0.08 ± 0.45		−0.08 ± 0.46	

Note: * Significant difference between two groups (*p* < 0.05).

## Data Availability

The data presented in this study are available on request from the corresponding author. The data are not publicly available due to protecting participant confidentiality.
